# Risk Factors of Ischemic Stroke in Young Adults: A Chinese Single-Center Study

**DOI:** 10.3389/fneur.2022.874770

**Published:** 2022-05-18

**Authors:** Mingyu Tang, Guangsong Han, Ming Yao, Bin Peng, Yicheng Zhu, Lixin Zhou, Jun Ni

**Affiliations:** Department of Neurology, State Key Laboratory of Complex Severe and Rare Diseases, Peking Union Medical College Hospital, Chinese Academy of Medical Sciences and Peking Union Medical College, Beijing, China

**Keywords:** ischemic stroke in young adults, etiology, risk factors, stroke of undetermined etiology, IPSS category

## Abstract

**Introduction:**

The etiology and risk factor profile of ischemic stroke in young adults are different from those in older patients. However, current etiological classifications are more applicable for the older adults, posing a challenge to the diagnosis of young patients with ischemic stroke. In this study, we applied a modified risk factor categorization previously used in the International Pediatric Stroke Study (IPSS) to describe the risk factor profiles of Chinese young patients with ischemic stroke and explore the sex and age differences in the distribution of risk factors.

**Methods:**

This is a single-center retrospective study. Patients aged 18–50 years with a first-ever ischemic stroke admitted to the Peking Union Medical College Hospital between 2013 and 2020 were consecutively included. The risk factors of patients were collected and divided into 10 categories according to the modified IPSS criteria and the sex and age differences were explored.

**Results:**

A total of 538 patients were enrolled in this study. The median age was 39 years and 62.6% were men. At least one IPSS risk factor category was identified in the 93.3% of all patients. The most common IPSS subtype was atherosclerosis-related risk factors (61.7%), followed by prothrombotic states (27.3%), chronic systemic conditions (24.7%), arteriopathy (16.2%), and cardiac disorders (10.4%). Chronic systemic conditions were more prevalent in patients aged <35 years (34.0 vs. 19.6%, *p* < 0.05) and women (43.3 vs. 13.6%, *p* < 0.0001). Atherosclerosis-related risk factors were more dominant in patients aged ≥35 years (72.6 vs. 41.9%, *p* < 0.0001) and men (77.2 vs. 35.8%, *p* < 0.0001).

**Conclusions:**

The IPSS classification might be a potential tool to better identify the risk factors of ischemic stroke in young adults.

## Introduction

The incidence of ischemic stroke in young adults has increased by 40% in recent decades (1, 2), causing over two million young patients with stroke yearly (1, 3). Epidemiological studies have revealed an increasing proportion of ischemic stroke in young adults, which accounts for 10–20% of total ischemic stroke events (4). In contrast with the elderly, young patients with stroke have a longer life expectancy, leading to high health care costs, severe labor productivity loss, and heavy economic burdens on individuals, families, and society (3). Therefore, more efforts should be made for the identification of risk factors (RFs) and the pathogenesis of stroke in young adults to improve the management and prevention (5).

Compared with stroke in the older adults, ischemic stroke in young adults has its own features. First, the causes and risk factors are various and relatively rare, such as artery dissections, autoimmune diseases, and illicit drug use, suggesting the need for specific investigation strategies (6). Second, there are age and sex differences in risk factor distribution. For example, pregnancy and the use of contraceptive drugs are only related to ischemic stroke in young women (7, 8), and among patients aged over 35 years, the prevalence of vascular risk factors rises remarkably than in the younger population (4). Therefore, the sex-specific and age-related variations in risk factor distribution require an individualized diagnostic workup.

However, the current classifications of ischemic stroke, such asthe Trial of Org 10,172 in Acute Stroke Treatment (TOAST) criteria, are developed mainly for stroke in older people. According to some large-scale studies of the etiology of ischemic stroke in young adults, a large proportion of patients were classified as other determined etiology or undetermined etiology (9–13), which is not conducive to rapid etiological identification and poses a challenge on the treatment and prevention of ischemic stroke in young adults. In addition, when TOAST classification is used for etiological diagnosis, strategy for secondary prevention is usually carried out mainly to prevent the recurrent thrombus formation without considering treating other potential mechanisms. However, a part of young patients with ischemic stroke may have other potential risk factors or pathogenic mechanisms. Based on these points, TOAST classification is not applicable to all young patients with stroke.

Therefore, increasing studies are exploring the new classifications of risk factors and the mechanism of stroke in young adults. Risk factor profiles for pediatric ischemic stroke have been proved to be distinct from those in older adults, and in 2011, researchers used a new classification which divided the presumptive risk factors of childhood ischemic stroke into ten categories in the International Pediatric Stroke Study (IPSS) (14). Since the risk factor spectrum of pediatric ischemic stroke partly overlaps with ischemic stroke among young adults, in 2018, the Follow-Up of Transient ischemic attack and stroke patients and Unelucidated Risk factor Evaluation (FUTURE) study first tried to categorize the risk factors of ischemic stroke in young adults by IPSS criteria and found that at least one IPSS category could be identified in 94% of all participants and in 88% of patients classified into the stroke of unknown etiology according to TOAST criteria. Therefore, they concluded the modified risk factor inventory based on pediatric findings, providing a valuable starting point for the development of a young stroke specific classification system (15). However, to confirm its feasibility, more investigations conducted in more ethnic groups and geographical regions are required.

In this study, we aimed to identify the risk factor distribution of ischemic stroke in young adults according to the IPSS classification in a large-scale Chinese cohort. The second aim was to explore the variations in risk factor distribution between different genders and age groups, which might lead to the better identification of potential mechanism, clinical management, and the prevention of ischemic stroke in young adults.

## Methods

### Study Participants

This study is a single-center retrospective study. Patients aged 18–50 years with a first-ever ischemic stroke admitted to the Peking Union Medical College Hospital between 2013 and 2020 were consecutively included in the study. Ischemic stroke was defined as an acute focal or diffuse neurological deficit persisting for at least 24 h, referable to the territory of cerebral arteries and confirmed by the neuroimaging (brain computed tomography [CT] or magnetic resonance imaging [MRI]). Patients were excluded if they were diagnosed with transient ischemic attack (TIA), hemorrhagic stroke, or cerebral venous system thrombosis. The study was approved by the Ethics Committees of Peking Union Medical College Hospital, approval number JS-1281.

### Study Design

The following information of all patients were extracted from the electronic medical records database of Peking Union Medical College Hospital and were documented structurally: (1) the demographic data of patients; (2) clinical presentations; (3) medical history; (4) laboratory findings, such as complete blood count, blood chemistry (transaminases, creatinine, urea nitrogen, electrolytes, fasting glucose, hemoglobin A1c, high-density lipoprotein cholesterol, low-density lipoprotein cholesterol, triglycerides, C-reactive protein, and homocysteine), prothrombin time, and activated partial thromboplastin time; (5) neuroimaging, such as CT and MRI; (6) vascular imaging, such as cervical vascular ultrasound, transcranial Doppler (TCD), resonance angiography (MRA), computed tomography angiography (CTA), and/or digital subtraction angiography (DSA); (7) cardiac examinations, such as 12-lead electrocardiography and transthoracic echocardiography; and (8) other examinations were carried out depending on the patient-specific conditions, such as screening for thrombophilia (protein C and S and antithrombin), antiphospholipid antibodies (anticardiolipin, anti-β2 glycoprotein 1 antibody, and lupus anticoagulant), and screening for autoimmune diseases (antinuclear antibody, anti-extractable nuclear antigen antibody, and antineutrophil cytoplasmic antibodies), 24-h Holter monitoring, transesophageal echocardiography, and bubble-TCD.

Etiology was classified according to TOAST criteria (16) by experienced neurologists into the following subtypes: large-artery atherosclerosis, cardioembolism, small-vessel occlusion, stroke of other determined etiology, and the stroke of undetermined etiology.

The presumptive stroke risk factors were collected and divided into nine categories according to the IPSS definitions: (1) arteriopathy: any arterial abnormality on vascular imaging other than isolated vessel occlusion; (2) cardiac disorders: either a history of chronic cardiac disorder or when detected on electrocardiograph or echocardiography during the analysis of stroke; (3) chronic systemic conditions (CSCs): a condition or disease with known changes in coagulation or vascular structure, such as autoimmune disease (e.g., systemic lupus erythematosus, Sjogren's syndrome, and vasculitis), genetic disorder, hematological disease, inflammatory or immune system disorder, oncological disease, and use of oral contraceptives; (4) acute systemic conditions (ASCs): any acute condition that leads to systemic disturbances, e.g., hypotension, shock, <72 h after surgery; (5) prothrombotic states (PTSs): a known disease in coagulation or found on laboratory testing, such as hyperhomocysteinemia, antiphospholipid syndrome, protein C/S deficiency; (6) acute head and neck disorders (AHNDs): an acute disease, surgery or trauma localized in the head or neck region; (7) chronic head and neck disorders (CHNDs): a chronic disease localized in the head or neck region, e.g., tumor, aneurysm, or migraine; (8) infection; and (9) atherosclerosis-related RFs: either a history of a risk factor (mentioned in the medical history or the use of medication) or detected during the analysis of the stroke, e.g., hypertension, diabetes mellitus, hyperlipemia, smoking, and alcoholism (14). All diseases were diagnosed depending on the clinical manifestations and diagnostic workup according to the criteria of guidelines. Furthermore, pregnancy was added as the tenth risk factor category according to the modified version in the FUTURE study (15), considering that the pregnancy and postpartum period (<6 weeks) is a specific risk factor for ischemic stroke among young women. A patient could be divided into several categories which are not exclusive.

The proportion of each TOAST subtype and IPSS category was calculated as a whole and separately stratified by sex and age to explore the differences in risk factor distribution between different genders and age groups. Considering that vascular risk factors increases more pronounced in the population aged 35 years and older (4), and the age of 35 years have already been chosen as a cut-off value to perform stratified analysis in the many previous studies of young ischemic stroke (9, 10, 13), we used 35 years as a cut-off value. Moreover, we applied the IPSS categorization additionally in patients divided into the stroke of undetermined etiology to explore its feasibility.

### Statistical Analyses

Means or medians were used to show the average levels of quantitative data with or without a normal distribution, respectively. The *T*-test or rank-sum test was used to compare quantitative variables between groups for data with a normal or non-normal distribution. Chi-square or Fisher's exact test was used to compare categorical variables between different groups. SAS 9.4 was used for statistical analyses and the value of *p* < 0.05 was considered statistically significant.

## Results

### Baseline Information

In total, 538 patients were enrolled in this study. The detailed demographic data are summarized in Table 1. The median age was 39 years (interquartile range [IQR]: 31–45 years), and 62.6% were men. Women were younger than men at stroke onset (37 vs. 39, *p* < 0.05). Besides, the proportion of men was larger in patients ≥35 years than patients <35 years (67.4 vs. 53.9%, respectively, *p* < 0.05).

**Table 1:** Demographic data and TOAST classification.

**Characteristics**	**Total (*****n*** **= 538)**		**Sex group**				***p* value**	**Age group**				***p*-value**
			**Male (*****n*** **= 337)**		**Female (*****n*** **= 201)**			**<35 years (*****n*** **= 191)**		**≥35 years (*****n*** **= 347)**		
**Age (y)**	39	(31,45)	39	(33,45)	37	(30,44)	0.0039^*^	-	-	-	-	-
**Male**, ***n*** **(%)**	337	(62.6)	-	-	-	-	-	103	(53.9)	234	(67.4)	0.0019^*^
**TOAST classification**, ***n*** **(%)**												
Large-artery atherosclerosis	185	(34.4)	149	(44.2)	36	(17.9)	<0.0001^*^	42	(22.0)	143	(41.2)	<0.0001^*^
Cardioembolism	32	(5.9)	19	(5.6)	13	(6.5)	0.6939	17	(8.9)	15	(4.3)	0.0317^*^
Small-vessel occlusion	36	(6.7)	33	(9.8)	3	(1.5)	0.0002^*^	4	(2.1)	32	(9.2)	0.0015^*^
Other determined etiology	214	(39.8)	97	(28.8)	117	(58.2)	<0.0001^*^	103	(53.9)	111	(32.0)	<0.0001^*^
Undetermined etiology	71	(13.2)	39	(11.6)	32	(15.9)	0.1495	25	(13.1)	46	(13.3)	0.9562

*TOAST, Trial of Org 10,172 in Acute Stroke Treatment*.

* *p < 0.05*.

### TOAST Subtypes

The distribution of etiologic subtypes classified by the TOAST criteria is shown in Table 1. The stroke of other determined etiology was the most prevalent stroke subtype (39.8%) in our cohort, followed by large-artery atherosclerosis (34.4%), stroke of undetermined etiology (13.2%), small-vessel occlusion (6.7%), and cardioembolism (5.9%).

The Trial of Org 10,172 in Acute Stroke Treatment subtypes differed distinctly between men and women. Men were identified much more frequently in large-artery atherosclerosis (44.2 vs. 17.9%, *p* < 0.0001) and small-vessel occlusion (9.8 vs. 1.5%, *p* < 0.05) compared with women. In contrast, the stroke of other determined etiology was more common among women (58.2 vs. 28.8%, *p* < 0.0001).

The predominant etiologic subtype varied with age. Large-artery atherosclerosis (41.2 vs. 22.0%, *p* < 0.0001) and small-vessel occlusion (9.2 vs. 2.1%, *p* < 0.05) were more prevalent in patients aged ≥35 years than in patients aged <35 years. On the other hand, the stroke of other determined etiology (53.9 vs. 32.0%, *p* < 0.0001) and cardioembolism (8.9 vs. 4.3%, *p* < 0.05) were more common in patients aged <35 years than in patients aged ≥35 years.

### Risk Factors Categorized According to IPSS Criteria

Table 2 lists the detailed IPSS categories and demonstrates the prevalence of risk factors categorized according to IPSS criteria. Among 538 patients, 93.3% of them had at least one IPSS category. Besides atherosclerosis-related RFs (61.7%), the most common IPSS subtype was prothrombotic states (27.3%), followed by chronic systemic conditions (24.7%), arteriopathy (16.2%), and cardiac disorders (10.4%). The prevalence rates of other categories were all <5%.

**Table 2:** The IPSS risk factor category.

**IPSS risk factor category, *n* (%)**	**Total (*****n*** **= 538)**		**Sex group**				***p* value**	**Age group**				***p*-value**
			**Male (*****n*** **= 337)**		**Female (*****n*** **= 201)**			**<35 years (*****n*** **= 191)**		**≥35 years (*****n*** **= 347)**		
**≥1 category**	502	(93.3)	320	(95.0)	182	(90.5)	0.0478^*^	174	(91.1)	328	(94.5)	0.1282
**Arteriopathy**	87	(16.2)	47	(13.9)	40	(19.9)	0.0696	39	(20.4)	48	(13.8)	0.0471^*^
Moyamoya disease	38	(7.1)	12	(3.6)	26	(12.9)	<0.0001^*^	13	(6.8)	25	(7.2)	0.8630
Arterial dissection	26	(4.8)	21	(6.2)	5	(2.5)	0.0501	17	(8.9)	9	(2.6)	0.0011^*^
Hereditary vascular disease^a^	11	(2.0)	7	(2.1)	4	(2.0)	1.0000	6	(3.1)	5	(1.4)	0.3100
Vasculitis secondary to infections	4	(0.7)	2	(0.6)	2	(1.0)	0.9954	1	(0.5)	3	(0.9)	1.0000
Reversible cerebral vasoconstriction syndrome	4	(0.7)	2	(0.6)	2	(1.0)	0.9954	0	(0)	4	(1.2)	0.3346
Fibromuscular dysplasia	3	(0.6)	2	(0.6)	1	(0.5)	1.0000	2	(1.0)	1	(0.3)	0.5987
Primary angiitis of central nervous system	1	(0.2)	1	(0.3)	0	(0)	1.0000	0	(0)	1	(0.3)	1.0000
**Cardiac disorders**	56	(10.4)	31	(9.2)	25	(12.4)	0.2340	27	(14.1)	29	(8.4)	0.0357^*^
Patent foramen ovale	14	(2.6)	10	(3.0)	4	(2.0)	0.4909	10	(5.2)	4	(1.2)	0.0104^*^
Endocarditis	13	(2.4)	6	(1.8)	7	(3.5)	0.3403	7	(3.7)	6	(1.7)	0.2688
Valvulopathy	8	(1.5)	2	(0.6)	6	(3.0)	0.0644	4	(2.1)	4	(1.2)	0.6233
Previous cardiac history	7	(1.3)	4	(1.2)	3	(1.5)	1.0000	2	(1.0)	5	(1.4)	1.0000
Atrial fibrillation	8	(1.5)	3	(0.9)	3	(1.5)	0.8264	3	(1.6)	3	(0.9)	0.7510
Myocardiopathy	4	(0.7)	3	(0.9)	1	(0.5)	1.0000	0	(0)	4	(1.2)	0.3346
Other kinds of arrhythmia	3	(0.6)	1	(0.3)	2	(1.0)	0.6500	0	(0)	3	(0.9)	0.4942
Congenital heart disease	2	(0.4)	1	(0.3)	1	(0.5)	1.0000	2	(1.0)	0	(0)	0.1256
Other^b^	13	(2.4)	6	(1.8)	7	(3.5)	0.3403	5	(2.6)	8	(2.3)	1.0000
**Chronic systemic conditions**	133	(24.7)	46	(13.6)	87	(43.3)	<0.0001^*^	65	(34.0)	68	(19.6)	0.0002^*^
Autoimmune disease	81	(15.1)	25	(7.4)	56	(27.9)	<0.0001^*^	49	(25.7)	32	(9.2)	<0.0001^*^
Hematological disorder	33	(6.1)	15	(4.5)	18	(9.0)	0.0352^*^	9	(4.7)	24	(6.9)	0.3079
Oncological disease	16	(3.0)	2	(0.6)	14	(7.0)	<0.0001^*^	2	(1.0)	14	(4.0)	0.0509
Hyperthyroidism	11	(2.0)	4	(1.2)	7	(3.5)	0.1323	7	(3.7)	4	(1.2)	0.0985
Oral contraceptive pill	3	(0.6)	0	(0)	3	(1.5)	0.0988	2	(1.0)	1	(0.3)	0.5987
Inflammatory disease	2	(0.4)	1	(0.3)	1	(0.5)	1.0000	2	(1.0)	0	(0.0)	0.1256
Other^c^	5	(0.9)	2	(0.6)	3	(1.5)	0.5572	4	(2.1)	1	(0.3)	0.1053
**Prothrombotic states**	147	(27.3)	109	(32.3)	38	(18.9)	0.0007^*^	53	(27.7)	94	(27.1)	0.8696
Hyperhomocysteinemia	116	(21.6)	102	(30.3)	14	(7.0)	<0.0001^*^	35	(18.3)	81	(23.3)	0.1756
Antiphosholipid syndrome	32	(5.9)	12	(3.6)	20	(10.0)	0.0024^*^	16	(8.4)	16	(4.6)	0.0772
Protein C or S deficiency	2	(0.4)	0	(0)	2	(1.0)	0.1391	2	(1.0)	0	(0.0)	0.1256
Acquired thrombophilia	1	(0.2)	0	(0)	1	(0.5)	0.3736	1	(0.5)	0	(0.0)	0.3550
Hereditary thrombophilia	1	(0.2)	0	(0)	1	(0.5)	0.3736	1	(0.5)	0	(0)	0.3550
**Acute systemic disorders**	16	(3.0)	7	(2.1)	9	(4.5)	0.1128	3	(1.6)	13	(3.7)	0.1551
<72h after surgery	11	(2.0)	4	(1.2)	7	(3.5)	0.1323	3	(1.6)	8	(2.3)	0.7964
Shock	5	(0.9)	3	(0.9)	2	(1.0)	1.0000	0	(0)	5	(1.4)	0.2310
**Chronic head and neck disorders**	14	(2.6)	6	(1.8)	8	(4.0)	0.1211	7	(3.7)	7	(2.0)	0.3866
Aneurysm	6	(1.1)	2	(0.6)	4	(2.0)	0.2856	1	(0.5)	5	(1.4)	0.5888
Migraine	5	(0.9)	2	(0.6)	3	(1.5)	0.5572	4	(2.1)	1	(0.3)	0.1053
Brain tumor	2	(0.4)	0	(0)	2	(1.0)	0.1391	0	(0)	2	(0.6)	0.5412
Rarry-Romberg Syndrome	1	(0.2)	1	(0.3)	0	(0)	1.0000	1	(0.5)	0	(0)	0.3558
**Acute head and neck disorders**	6	(1.1)	0	(0)	6	(3.0)	0.0057^*^	3	(1.6)	3	(0.9)	0.7510
Head or neck surgery	6	(1.1)	0	(0)	6	(3.0)	0.0057^*^	3	(1.6)	3	(0.9)	0.7510
**Infection**	7	(1.3)	3	(0.9)	4	(2.0)	0.4865	2	(1.0)	5	(1.4)	1.0000
**Pregnancy related**	1	(0.2)	0	(0)	1	(0.5)	-	1	(0.5)	0	(0)	0.3558
**≥1 atherosclerosis-related risk factor**	332	(61.7)	260	(77.2)	72	(35.8)	<0.0001^*^	80	(41.9)	252	(72.6)	<0.0001^*^
Hypertension	251	(46.7)	193	(57.3)	58	(28.9)	<0.0001^*^	51	(26.7)	200	(57.6)	<0.0001^*^
Diabetes mellitus	114	(21.2)	79	(23.4)	35	(17.4)	0.0978	20	(10.5)	94	(27.1)	<0.0001^*^
Hyperlipemia	142	(26.4)	112	(33.2)	30	(14.9)	<0.0001^*^	36	(18.8)	106	(30.5)	0.0032^*^
Smoking	171	(31.8)	151	(44.8)	20	(10.0)	<0.0001^*^	43	(22.5)	128	(36.9)	0.0006^*^
Alcoholism	83	(15.4)	79	(23.4)	4	(2.0)	<0.0001^*^	11	(5.8)	72	(20.7)	<0.0001^*^

*IPSS, International Pediatric Stroke Study*.

a *Including cerebral autosomal dominant arteriopathy with subcortical infarcts and leukoencephalopathy (CADASIL, n = 7), cerebral autosomal recessive arteriopathy with subcortical infarcts and leukoencephalopathy (CARASIL, n = 2) and Fabry disease (n = 2)*.

b *Including chronic cardiac failure (n = 3), acute cardiac failure (n = 2), cardiac tumor (n = 2), cryptogenic mass in aortic root (n = 2), pulmonary arterial hypertension with cardiac failure (n = 1), myocardial infarction (n = 1), aortic dissection (n = 1), and cardiac hydatid cysts (n = 1).*

c *Including multicentric Castleman disease (n = 1), nephrotic syndrome (n = 1), chronic renal failure (n = 1), hypothyroidism (n = 1), and adrenocortical hypofunction (n = 1).*

* *p < 0.05*.

There were significant differences in the IPSS category distribution between men and women. Men were more likely to be classified as prothrombotic states than women (32.3 vs. 18.9%, *p* < 0.05). In this category, hyperhomocysteinemia was more dominant in men (30.3 vs. 7.0%, *p* < 0.0001) while antiphospholipid syndrome was more common in women (10.0 vs. 3.6%, *p* < 0.05). Chronic systemic conditions were more commonly present among female patients than male patients (43.3 vs. 13.6%, *p* < 0.0001). In this category, autoimmune disease (27.9 vs. 7.4%, *p* < 0.0001), hematological disorder (9.0 vs. 4.5%, *p* < 0.05), and oncological disease (7.0 vs. 0.6%, *p* < 0.0001) were more prevalent in women. AHNDs were more reported in women (3.0 vs. 0, *p* < 0.05) while atherosclerosis-related RFs were more predominant in men (77.2 vs. 35.8%, *p* < 0.0001).

The distribution of IPSS category varied between different age groups. Patients <35 years of age were more likely to be classified as arteriopathy (20.4 vs. 13.8%, *p* < 0.05), cardiac disorders (14.1 vs. 8.4%, *p* < 0.05), and chronic systemic conditions (34.0 vs. 19.6%, *p* < 0.05) than patients ≥35 years. In contrast, atherosclerosis-related RFs were more dominant in patients ≥35 years (72.6 vs. 41.9%, *p* < 0.0001). Besides, autoimmune disease (25.7 vs. 9.2%, *p* < 0.0001), artery dissection (8.9 vs. 2.6%, *p* < 0.05), and patent foramen ovale (5.2 vs. 1.2%, *p* < 0.05) were more present in younger patients.

In 71 patients classified as the stroke of undetermined etiology according to TOAST criteria, 74.6% were found to have at least one risk factor with IPSS approach. Besides atherosclerosis-related RFs (49.3%), the most reported subtype was prothrombotic states (18.3%), followed by cardiac disorders (15.5%) and chronic systemic conditions (14.1%). The detailed information is demonstrated in Table 3.

**Table 3:** The IPSS category in stroke of undetermined etiology.

**IPSS category**	***n***	**(%)**
≥1 category	53	(74.6)
Atherosclerosis-related risk factors	35	(49.3)
Prothrombotic states	13	(18.3)
Cardiac disorders	11	(15.5)
Chronic systemic conditions	10	(14.1)
Acute systemic conditions	5	(7.0)
Infection	3	(4.2)
Chronic head and neck disorders	3	(4.2)
Acute head and neck disorders	2	(2.8)

*IPSS, International Pediatric Stroke Study*.

### Modifiable Cardiocerebrovascular Risk Factors

Table 2 summarizes the prevalence of classical cerebrovascular risk factors in our cohort. At least one modifiable cerebrovascular risk factor was found in 61.7% of all patients. We found hypertension in 251 of 538 patients (46.7%), smoking in 171 (31.8%), hyperlipidemia in 142 (26.4%), diabetes mellitus in 114 (21.2%), and alcoholism in 83 (15.4%).

On average, men tended to have more modifiable cerebrovascular risk factors than women (2 vs. 0, *p* < 0.0001). Hypertension (57.3 vs. 28.9%, *p* < 0.0001), hyperlipidemia (33.2 vs. 14.9%, *p* < 0.0001), smoking (44.8 vs. 10.0%, *p* < 0.0001), and alcoholism (23.4 vs. 2.0%, *p* < 0.0001) were found to be more significantly present in men than in women.

More modifiable cerebrovascular risk factors were found among patients aged 35 years or older than patients younger than 35 years (2 vs. 0, *p* < 0.0001). Hypertension (57.6 vs. 26.7%, *p* < 0.0001), diabetes mellitus (27.1 vs. 10.5%, *p* < 0.0001), hyperlipidemia (30.5 vs. 18.8%, *p* < 0.05), smoking (36.9 vs. 22.5%, *p* < 0.05), and alcoholism (20.7 vs. 5.8%, *p* < 0.0001) were more prevalent in patients ≥35 years.

## Discussion

This study is a large-scale cohort study in which we applied a risk factor categorization first used in the IPSS into the Chinese young patients with ischemic stroke. We identified at least one IPSS category in 93.3% of all patients, and 74.6% of patients classified as the stroke of undetermined etiology according to the TOAST criteria. Distinct gender and age differences in IPSS risk factor distribution were observed. Our findings suggest that the IPSS classification might be a potential supplement to better identify the risk factors of ischemic stroke in young adults and provide a basis for further understanding of stroke mechanism.

The IPSS categorization could be an effective supplement to the traditional pathogenesis classifications for ischemic stroke which, such as the TOAST criteria, are mainly designed for the older adults. It has been reported by several studies that a large proportion of young patients with ischemic stroke, approximately 15–50% (11–13), would be divided into the stroke of undetermined etiology if classified by the TOAST criteria, varying with the geographical regions and local medical resources. This suggests that current categorizations for ischemic stroke are not appropriate for young patients with stroke and might pose a challenge on the pathogenesis study and etiologic diagnosis. Moreover, present stroke classifications cannot guide the clinical management of stroke in young adults precisely, which are mainly extrapolated from the older adults (5) and often lead to a “one-size-fits-all” strategy of antithrombotic treatment rather than individualized treatment (15). Therefore, it is necessary to explore the potential risk factors and mechanisms of young adults with ischemic stroke and establish its specific classification approach to lead to rapid etiological identification and prevention strategies. In our study, at least one IPSS category was found in 93.3% of all patients and 74.6% of patients classified as the stroke of undetermined etiology, indicating the underlying feasibility of this novel risk factors categorization.

It is worth noting that although rare causes and undetermined etiology account for a large proportion of stroke in young adults, atherosclerosis and modifiable vascular risk factors are becoming increasingly prevalent among young patients with stroke (17–19). In our study, large-artery atherosclerosis was the second commonly reported TOAST subtype, and atherosclerosis-related RFs were observed in over 60% of all patients, especially in men and patients ≥35 years of age. This emphasizes the necessity of the strict management of traditional cerebrovascular risk factors in young patients with ischemic stroke, even though in those who have other causes.

Besides atherosclerosis-related RFs, the dominant IPSS subtypes in our cohort were prothrombotic states, chronic systemic conditions, arteriopathy, and cardiac disorders, which accounted for over 10%, respectively. Prothrombotic states are much more prevalent in our cohort compared with the FUTURE study due to a relatively high percentage of hyperhomocysteinemia (21.6%) in our cohort, which is consistent with a previous Chinese epidemiological study (20). Hyperhomocysteinemia is widely acknowledged as a risk factor for both stroke and cardiac diseases (21). Its potential mechanism includes stimulating the platelet generation of thromboxane A2 (22), producing reactive oxygen species (ROS), deactivating epithelial nitric oxide (NO), inducing proliferation of intimal smooth muscle cells (23), and ultimately leading to a pro-thrombotic state. Hyperhomocysteinemia could be either an inherited or acquired disease. In previous studies, the prevalence of hyperhomocysteinemia in young patients with stroke varies with ethnic groups, from 7 to 16% in Caucasians to over 20% in Chinese (9–11). Though there are current knowledge gaps in the epidemiology of pathogenic genetic mutations among different races, the specific eating habits of some Chinese people, which leads to a deficiency of vitamin B (especially folate, B_6_, and B_12_), might be one of the causes of the relatively high prevalence.

There were obvious differences in the IPSS category distribution between male and female patients. Chronic systemic conditions and AHNDs were more prevalent among young women. By contrast, prothrombotic states and atherosclerosis-related RFs were found to be more among men. Moyamoya disease, antiphospholipid syndrome, autoimmune disease, hematological disorder, and oncologic disease were more common among women, while the prevalence of hyperhomocysteinemia in men was significantly higher. Some of those disparities, such as the variations in atherosclerosis-related RFs and autoimmune disease, are consistent with previous studies (24, 25). The underlying mechanism may include the cytoprotective properties of estrogen (26, 27) and sex differences in immune response (25). These differences shown by the IPSS profile suggest underlying etiological clues and re-emphasize the significance of sex-specific diagnostic strategies for young adults with ischemic stroke.

Moreover, the IPSS distribution significantly varied with age. In patients <35 years of age, non-atherosclerotic arteriopathy, cardiac disorder, and chronic systemic conditions were much more identified while atherosclerosis-related RFs were less frequently observed in contrast with relatively older patients. The age-related variations in the IPSS spectrum reveal the importance of different diagnostic work-up and the prevention projects of ischemic stroke in young adults according to age.

The strengths of our study are as follows. First, it was carried out in a tertiary Chinese hospital and the etiology and the risk factors of young patients with stroke were various, such as rare kinds of diseases. What' s more, the large-scale cohort provided a comprehensive risk factor profile of the Asian young patients with stroke. Finally, exploring the difference in the IPSS risk factor distribution between different age and sex groups lays a foundation for further pathogenesis research of ischemic stroke in young adults.

There are some limitations in our study. First, as the study was conducted in a top-level tertiary medical center in China, the selection bias should be taken into consideration. The prevalence of rare diseases might be overestimated and other subtypes of TOAST classification, such as small-vessel occlusion and cardiac embolism might be underestimated. Furthermore, information missing is an inevitable disadvantage of retrospective study. It might lead to a relatively low prevalence of some specific risk factors, such as migraine, which were likely to be neglected and not usually specifically documented in medical records. Additionally, some recruited patients might not perform complete investigations because of either economic or hospital stay constraints. Finally, it should be noted that the IPSS categorization is considered as a kind of presumptive risk factor categorization rather than an etiologic classification, as some of the IPSS categories might be just comorbidities or the complications of stroke. Therefore, careful differential diagnosis is required in clinical application, and the verification of causality is supposed to be focused in further research. The improvement and modification of IPSS categorization, for example, exploring a weighting points-scoring version or adopting more advanced workup into the diagnostic process, are expected in the future to make it more instructive in the clinical practice.

Overall, our study described the comprehensive risk factor profile of ischemic stroke among young Chinese adults according to a modified categorization initially designed for pediatric stroke, and explored the age and sex differences in risk factor distribution. At least one IPSS category was reported in 93.3% of all patients, and 74.6% of patients classified as the stroke of undetermined etiology according to the TOAST criteria, revealing more potential risk factors associated with other stroke mechanisms in the young than in the elderly. Meanwhile, the age-related variations and sex-specific disparities in risk factor distribution provide clues for future etiologic study of adult early-onset ischemic stroke. Our findings suggest the potential feasibility of IPSS categorization in clinical practice. We hope our study could lay a foundation for future studies conducted across different regional sites and ethnic races, and ultimately develop a specialized etiological classification for young patients with stroke, leading to better pathogenetic understanding and more individualized treatment strategies.

## Data Availability Statement

The raw data supporting the conclusions of this article will be made available by the authors, without undue reservation.

## Ethics Statement

The studies involving human participants were reviewed and approved by Ethics Committees of Peking Union Medical College Hospital. The patients/participants provided their written informed consent to participate in this study.

## Author Contributions

BP, YZ, LZ, and JN: study concept and design. MT, MY, and GH: data collection and analysis. MT: manuscript drafting. LZ and JN: critical revision of the manuscript for intellectual content. All authors contributed to the article and approved the submitted version.

## Funding

This research was supported by the Central Public-interest Scientific Institution Basal Research Fund of Chinese Academy of Medical Sciences (#2021-RW320-003).

## Conflict of Interest

The authors declare that the research was conducted in the absence of any commercial or financial relationships that could be construed as a potential conflict of interest.

## Publisher's Note

All claims expressed in this article are solely those of the authors and do not necessarily represent those of their affiliated organizations, or those of the publisher, the editors and the reviewers. Any product that may be evaluated in this article, or claim that may be made by its manufacturer, is not guaranteed or endorsed by the publisher.
